# Interprofessional Teamwork to Promote Health: First-Time Parents' Experiences of a Combined Home Visit by Midwife and Child Health Care Nurse

**DOI:** 10.3389/fped.2022.717916

**Published:** 2022-03-03

**Authors:** Katarina Sjögren Forss, Elisabeth Mangrio, Lisa Hellström

**Affiliations:** ^1^Department of Care Science, Faculty of Health and Society, Malmö University, Malmö, Sweden; ^2^Department of School Development and Leadership, Faculty of Education and Society, Malmö University, Malmö, Sweden

**Keywords:** child health care, health promotion, interprofessional teamwork, interviews, parenthood, nursing, midwifery

## Abstract

**Background:**

To achieve the requisites for a child's healthy development and to reduce health inequalities, it is important to promote health initiatives at an early stage in a child's life and to include the parents. Home visits by healthcare professionals have been found to have positive health effects for both the baby and the parents. From an extended home visit programme in Sweden, our aim was to illuminate first-time parents' experience of a home visit conducted by a midwife and a child health care nurse 1–2 weeks postnatal.

**Methods:**

Data was collected by interviews (*n* = 13) with first-time parents. The transcribed texts were analyzed using inductive content analysis.

**Results:**

The participants' experiences could be understood from the two themes, *A trust in the professionals* and *Feeling safe as a new parent*. The participants experienced that the midwives and the child health care nurses complemented each other and appreciated to get knowledge and information from both professions. In their own home, they felt secured and relaxed, and the professionals could help them provide a safe home environment for the child.

**Conclusion:**

By meeting both professionals at the same time and in their own home, the participants experienced that the needs of the baby and their needs and concerns as new parents were included and supported.

## Introduction

The period of early childhood has been identified as a huge driver of inequalities in health (1) and has been shown to be the most crucial period of lifespan development (2). As inequalities in health is well established and both social and structural factors have been found to have a huge influence in early childhood, it is essential to implement various health promotion initiatives to reduce these inequalities (3). Health promotion initiatives have also been found valuable for first-time parents, as the transition to parenthood is a major and challenging life-changing event for most couples. Therefore, interventions to strengthen their relationship, address their feelings of parental anxiety, and provide support are important (4).

In previous studies (5–8), home visits by healthcare professionals have been found to have positive health effects among first-time parents in both short-term and long-term perspectives. The studies have also shown that these visits have positive health effects on the child's physical and mental health. The child also experiences reduced vulnerability and fewer language delays. In Sweden, child healthcare services (CHC) work to promote children's physical, psychological and social health by promoting good health and development (9). This work is in line with the World Health Organization's (10) initiative that promotes care for early childhood development. In addition to a stable, supportive and responsive environment for the young child, this initiative emphasizes good health and nutrition, protecting the child from threats, and giving the child possibilities for early learning.

This work is mainly performed by CHC nurses, starting with a home visit when the child is about 1 week old, and then followed by regular visits at CHC centers. A Swedish intervention study of an extended home-visit programme that included six home visits by a CHC nurse and a parental advisor from social services resulted in better vaccination coverage of the measles, mumps and rubella vaccine and fewer hospital visits among the children whose parents participated as (11). Also, fathers who participated stated that the programme met their need for more knowledge and greater confidence as a parent (12). Therefore, it is reasonable to continue and extend the concept and also focus more on interprofessional teamwork, as interprofessional collaborative practice is one of the priorities for maternal and CHC services worldwide (13).

As a result, the programme, *Growing Safely*, was suggested and funded by Region Scania in Sweden with the aim of contributing to equal health development in the population by an extended home-visit programme to first-time parents focusing on socially deprived areas. The programme was interprofessional and included six home visits. A CHC nurse attended all visits, and on the first visit, a midwife also attended (regarding the other visits, parental advisors from social services attended three visits and a dental assistant attended one visit). The programme lasted for a period of 15 months. However, the current study only focuses on the first home visit that was carried out by the CHC nurse and the midwife when the newborn was about 1–2 weeks old to capture the experiences of a home visits programme during the transition to parenthood. This home visit focuses on, for example, the delivery, the health of the baby, breastfeeding, and the mother's and father's questions, concerns and feelings about their new role as a parent.

Among public health professionals, there is a consensus that midwives make an essential contribution to high-quality maternal and newborn care (14, 15). Regarding midwives, Renfrew et al. (16) support a system-level shift, going from a focus on identification and treatment of pathology to, instead, a focus on preventive and supportive care that could strengthen women's capabilities. Care should be tailored to their needs, with a focus on normal reproductive processes, and of course, access to care when needed. Midwifery is essential to this work, which requires effective interdisciplinary teamwork and integration across community settings (16). Ten Hoope-Bender et al. (17) agree that, to ensure continuity and quality of care by midwives, varied competencies and expertise should be brought together with interprofessional practice.

To achieve the requisites for the healthy development for a child and to reduce the existence of health inequalities, it is important to include the parents in any health-promoting initiatives at an early stage in life. Teamwork between different professionals has been found to be important in this work. To the best of our knowledge, however, little is known about the type of interprofessional teamwork taking place between midwives and CHC nurses with first-time parents. Therefore, the aim of this study was to illuminate first-time parents' experience of a home visit that was conducted by a midwife and a CHC nurse 1–2 weeks after the baby was born.

## Methods

This study is part of a larger intervention of home visits for first-time parents initiated by the Swedish association of local authorities and regions and the region of Scania. Four different CHC centers in the county of Scania volunteered to participate and were recruited for the intervention. Recruitment of first-time parents for the interviews were made by registered nurses at the four CHC centers who introduced the research study by providing an information letter and collecting phone numbers from interested participants during their first home visit. Interested participants were then contacted by phone by the researchers to book a time for an interview. The researchers reassured that the interested participants were informed both about the aim of the study as well as about confidentiality and their right to withdraw at any point without needing to give any explanation for doing so. They were also informed that the interviews would be tape-recorded. The participants could choose if they wanted the interview to be held in Swedish, English, or if needed, held with an interpreter who spoke their primary language. They could also choose if they wanted the interview to be conducted in their home or by telephone and time was also given for the participants to ask questions concerning the study.

### Data Collection and Participants

The study was conducted September 2019–January 2020. Data was collected by interviews that took place either at the participants' home (*n* = 2) or by telephone (*n* = 11) by the first and last author. The interviews were held in close proximity (2–7 days) to the first home visit. Data saturation was reached after 13 interviews. In three of the interviews, both parents participated, and in the other interviews, it was either the mother or the father who participated. The age range for the participating families were, for mothers, 15–33 (*M* = 26), and for fathers, 27–37 (*M* = 31). Nine of the interviews were conducted in Swedish, two in English, and two were conducted with the help of an interpreter. See Table 1 for the sociodemographic characteristics of participants. The interviews lasted ~15–30 mins and were tape recorded and transcribed before the analysis. The authors made short field notes after the interviews. One pilot interview was held to assure the validity of the questions according to the study's aim. No changes were warranted, and the pilot interview was included in the analysis. An interview guide used was developed for this study. It included questions to gain an understanding of why the parents decided to participate in the extended home-visit programme, what their expectations of the CHC services were, and also how they experienced the combined home visit by the midwife and CHC nurse. The interview guide is provided as Additional File 1 in Supplementary Material.

**Table 1:** Sociodemographic characteristics of participating first-time parents (*N* = 25).

	**Mother (*N* = 13)**	**Father (*N* = 12)**
**Age range**		
15–20	1	
21–24	3	
25–29	6	1
30–34	3	9
35–39		2
**Country of birth**		
Afghanistan	2	2
Croatia	1	1
Finland		1
Iraq	1	1
Kosovo	1	1
Palestine	1	
Somalia	1	1
Sweden	2	2
Syria	2	2
Ukraine	1	1
**Residence time in Sweden**		
Born in Sweden	3	3
1–2 years	4	2
3–5	2	3
10–12 years	2	3
Occupation	Dental hygienist (1), assistant nurse (2), student (1), biomedical analyst (1), unemployed (1), unclear (1), childcare worker (1), beauty worker (1), student (1), hotel (1)	Medical doctor (1), engineer (2), communicator (1), construction worker (1), unclear (3), waitor (1), quality manager (1)

### Analysis

The interviews were analyzed using qualitative content analysis, as described by Elo and Kyngäs (18). First, the transcripts were read numerous times to obtain an overall understanding of the data. Thereafter, meaning units of the text that correspond with the aim were condensed and coded manually. As a third step, the codes were interpreted and compared to find similarities and differences and then sorted into tentative themes without losing their content. The first and the last author separately read and analyzed the text during the process and then discussed it to enhance the best possible account of the meaning found in the texts. Comparisons were made with the context in each step of the analysis to verify the empirical base of the data. Finally, the authors agreed on two themes.

## Results

Two themes, a trust in the professionals and feeling safe as a new parent, were identified and considered to reflect the participants' experience. The identified sub-themes are in italic for clarity.

### A Trust in the Professionals

The theme, A trust in the professionals, reflected the importance of continuity with a midwife that the mother already knew, but also that the parents valued the interprofessional collaboration between the midwife and CHC nurse and to have the opportinity to get knowledge and much practical advice from both professions.

#### The Important Role of a Known Midwife

It was described as important to have the home visit by a midwife that the mother already knew. This aspect was highlighted both by those who knew the visiting midwife from pregnancy as well as from those who did not. The mothers who received a home visit by the midwife who had followed them during their pregnancy explained that this already established relationship was a very positive aspect. Both parents experienced it as positive that that they had previously met the midwife. The midwife would already have a whole picture of the family, which meant the new parents did not have to retell every experience of the pregnancy, thus risking leaving out something important. The mothers in particular experienced the midwife as “an old friend” and appreciated the opportunity to discuss both the delivery and any medical questions related to their body.

*It was very good. You feel safe that it's the same people that were with you during pregnancy from start to finish … this person knows me and all that happened during pregnancy*. (Interview 4).

Those who met a new midwife in the first home visit without having established a previous relationship experienced it as stressful, especially if the pregnancy had been complicated. One mother also explained that this affected the interplay between the midwife and the CHC nurse because they did not have all the information needed to create a feeling of safety.

*The CHC nurse had forgotten to tell that I have been sick after the delivery and that I'd been admitted to the hospital and couldn't … yes, because they asked different questions if I could go out with my children … my child … and things like that. But I can't walk any long distances, and I'm in pain, so … They listened very carefully, and they gave tips … but it was the interplay between them … not between me and them*. (Interview 8).

#### The Interprofessional Knowledge Base of the Midwife and CHC Nurse

The parents expressed that the home visit fulfilled their needs as first-time parents and that the combined interprofessional knowledge base of the midwife and the CHC nurse made them feel secure. The added visits in the extended home-visits programme was also seen as positive when one has many questions and concerns.

*Yes, then you know that if it's not something that isn't really acute you can… you may have some question, then you know that, yes, they will come in two, three days or four days. Then we can wait until they come. You feel safe, as I said, that they are close at hand*. (Interview 6).

Further, the expertise and knowledge of the CHC nurse about children and their development was expressed as reassuring in regard to concerns about their baby. This included breastfeeding, stomach pain, weight gain and the sleeping habits of their newborn baby. The interprofessional knowledge of the midwife and the CHC nurse complemented each other, and there was a perception of respect toward each other's field of knowledge. By meeting both professionals simultaneously, the parents felt that the needs of the whole family were included and supported. One of the mothers summarized the home visit:

*The CHC nurse knows everything about the baby. She provided me with breastfeeding support, and I could ask many questions about the baby. And the midwife has knowledge about my body that the CHC nurse doesn't know, and those questions couldn't have been answered if the CHC nurse did the home visit alone*. (Interview 4).

### Feeling Safe as a New Parent

In the theme, Feeling safe as a new parent, the availability of the midwife and CHC nurse was described as a central component and the participants also appreciated to have them visiting their home as they could help them to provide a safe and supportive environment for the child. Mothers and fathers described different needs as new parents and it was important that both had the possibility to discuss their specific concerns with both professionals.

#### Availability of the Midwife and CHC Nurse

The parents experienced the CHC nurse as very available, and this make them feel safe in their new role as parents. Many maintained further contacts with her after the first home visit and described it as easy to get in contact. As first-time parents, the mothers and fathers highly appreciated the availability of the two professionals, regarding them as important for meeting their needs and creating a sense of safety. It also emerged that the role of the two professionals complemented each other well. The sense of safety was also expressed as having experienced professionals so close at hand in their home.

*When they were visiting, it felt like you could ask right away, a little bit like that … because you are all alone with it all and it feels safe to have experienced women around you who know so much*. (Interview 5).

To create feelings of safety and availability in the home visit, it also emerged that it is important for the participating parents to be available by showing an interest and asking questions concerning the safety of their baby and how to create a strong relationship.

#### The Importance of a Safe Home Environment

It was seen as positive that the first contact with the CHC nurse was established in their home, as she could see how they lived. If something in the home were potentially dangerous for the baby, she could alert them to this. In addition, receiving the interprofessional team of the midwife and CHC nurse in their home environment made the parents feel secure and relaxed. The home environment provided a sense of security for the parents to take care of the newborn baby's basic needs, such as breastfeeding, and the mother's needs as a new mother.

*The baby is so small, it's cold outside … and I am bleeding quite a lot, so I need to be home and relax instead of being at the hospital and staying there—because they have explained to me that you will stay there and share a room with other women and things like that*. (Interview 4).

The opportunity to be at home was also expressed as the convenience of not having to go to the childcare center for the checkups.

#### Parents Different Needs

With the CHC nurse, the mothers and fathers appreciated discussing both concerns about their baby and their new and inexperienced role as parents.

*So, it's like this—it's the first child, had it been… so we have no experience at all. Had it been… the second child, then many questions that you have today you may already have the answer to for the next child. But when it comes to the first child, much is unknown*. (Interview 6).

The experience of the visit for the fathers gave a sense of safety, as they now had someone to talk to concerning their roles as a father, as a partner and as an individual. It could be concerns about how to help the mother when she is breastfeeding, how to comfort her when she is crying, or how to cope with his own feelings about fatherhood:

*I have some feeling that if I have some stress that I keep inside me, and then I don't have any … discussion with people. I think that then I will find the way (to talk about it), and the stress will go out*. (Interview 2).

For the fathers, it seemed important to receive information regarding medical issues and get confirmation that the baby was developing and acting “normal,” while the mothers were more focused on breastfeeding and how to get to know the child and establish a relationship. The parents agreed that the combination of both professionals gave the best possible support for them as first-time parents and were surprised by this in a positive way. One mother expressed that the interprofessional teamwork between the midwife and CHC nurse were good at including the father in the conversation by recognizing when he wanted to ask questions and actively involving him in the conversation.

*They sense that he also wants to be included, because sometimes they don't want to … maybe the father isn't participating, and they can sense that and think “okay,” but if they see that he wants to, that he has questions, of course, they will try to include him as much as possible*. (Interview 7).

## Discussion

The current study aimed to illuminate first-time parents' experience of a home visit conducted by a midwife and a CHC nurse 1–2 weeks after the baby was born. Their experiences could be understood from two themes, *A trust in the professionals* and *Feeling safe as a new parent*.

Our results showed that the first-time parents perceived that the interprofessional knowledge of the midwives and the CHC nurses complemented each other, and they also perceived mutual respect toward each other's field of knowledge. The parents felt that, by meeting both professionals simultaneously, the whole family's needs were included and supported. It was also understood as important to have the visit in their own home. This also made the parents feel secure, safe and relaxed and corroborate with findings from Olander et al. (19) reporting the home environment as a familiar non-clinical setting providing parents more time to build a relation to healthcare professionals. Also a narrative review of the literature focusing on key components of health visiting practice by Cowley et al. (20) defined home visits as a core characteristics of health visits as it made participants feeling more relaxed and resulted in a better use of services.

In contrast to previous studies (21, 22) where parents have described their needs and health as somewhat neglected by the CHC nurses, the parents in our study experienced that their needs were sufficiently recognized. This may be explained by the possibility that the parents in our study had the opportunity to meet two professionals who filled different functions and needs for the parents. Previous studies have also shown that most mothers want to talk about their birth with a professional, and that midwives consider such a conversation important. However, only a few have the opportunity to do so, as it is not prioritized in clinical practice (23, 24). In our study, this was possible, and from our findings, we could see it was appreciated.

In addition, the relationship with a known midwife, who had followed the mother during pregnancy, was also important. These findings are in line with a previous study from Norway (25) showing the importance of having the home visit by a midwife who is already familiar with the woman's condition during pregnancy. From the findings in the Norwegian study (25), the authors concluded that having a discussion about the birth with an unfamiliar midwife was not productive for the woman, above all from emotional aspects Such a conclusion is not possible to draw from our study, as the study base is too small and most of the mothers had the home visit by the midwife who had followed her during her pregnancy. However, it is an important aspect to consider when planning such a home-visit program, especially if the pregnancy and delivery were complicated.

The results from our study confirm the findings from previous research (16, 17) showing the need to increase the availability and accessibility of the midwives. It also confirms the finding that midwives who work in an interdisciplinary way improve the health of both mothers and infants. We know that to promote health for both parents and infants (9), is it important for them to be followed closely, especially at an early stage in the newborn's life. It is also well known that women are at increased risk for post-partum depression (9) after the birth of their child. Many studies (26–28) have examined the risk factors and adverse effects of becoming a parent in women; however, the condition is less understood in men (29). The perinatal mental health of men has presently not been given the same importance as it has for women even if research shows the need of first-time fathers to have a professional to both discuss their mental health as well as concerns about the baby with (30). The CHC nurses and the midwives in our study visited the families together soon after the birth, and this could have beneficial effects in observing and supporting both parents, who may have an increased risk for depression in the postpartum period. Recognizing and treating parents' postpartum depression can improve the quality of life for the whole family and decrease the risk of emotional and social problems for the child and thus is an important aspect of health promotion. Our findings also showed that the fathers appreciated talking to a professional about their concerns from the perspectives of a father, husband and individual. This made the father visible as a parent rather than just a partner, which, in previous research, was found to be a common experience among fathers regarding pregnancy, birth and maternity care (31). First-time fathers have also reported feeling excluded by health professionals (30, 32, 33) resulting in seeking information for example about practical aspects of infant care from different online resources. However, this was described as uncertain and if accurate and reliable information was found (30).

In our study, we also found that the mothers and the fathers had different concerns about their new role as a parent. While the mothers were more concerned about breastfeeding and establishing a relation to the child, it was more important for the fathers to get confirmation that the baby was developing and acting “normal.” Similar findings about fathers' concerns and their need to have confirmed that everything is alright with the baby was described in a meta-synthesis by Brunstad et al. (34), who also reported that having emotional support from professionals empowered their feelings of confidence in the transition to fatherhood. This reinforces the potential importance of having different professionals involved in the home visit to give both parents enough space to discuss their concerns and support them in parenthood.

It is also important for both parents to have professionals with whom they can discuss any concerns and ask any questions they may have about parenthood. This is especially relevant today, as new parents often have less opportunities to learn about parenting from role models such as parents and extended family because living near to them is not as common as it were a few decades back (35). Migrant parents in particular often lack social support in the host country (36), and therefore, the support from this programme is important. However, the participants in our study also pointed out and understood the importance of their own involvement in the success of the programme.

Following a systematic literature review, Aquino et al. (37) found that both health visitors as well as midwives appreciated interprofessional collaborations above all the supportive aspects coming out from it, however challenges such as poor communication and understanding of each other's role were found and increased suppport to promote possibilities to collaborate is needed. A study from Austria about the struggle for interprofessional teamwork and collaboration to support and promote breastfeeding in maternity care (38) pointed out many differences between the approaches to childbirth and breastfeeding by nurses and midwives. For example, midwives experienced nurses as overprotective and also having a paternalistic approach when supporting breastfeeding; for example, midwives more often referred to breastfeeding as a natural process compared to nurses. Olander et al. (19) conclude that key components for a succesful interprofessional teamwork is characterized by flexibility, a common team approach and an early connection. From our findings we conclude that this interprofessional teamwork is important and that it worked out well from the perspective of the parents. However, we are well aware that differences between professions can be a barrier for interprofessional teamwork and more research is needed. In an upcoming study, we will interview the CHC nurses and midwives about their experiences of interprofessional teamwork.

### Strenghts and Limitations

The findings should be considered in the context of the study's limitations. They are based on a relatively small sample (*n* = 13) and relatively short interviews, which could have implications regarding its trustworthiness. The sample comprised people with diverse backgrounds and different experiences of healthcare from their country of origin. All participants had lived in Sweden for at least 1 year (most of them more) and all had during their pregnancy been visiting a midwife in Sweden. As all were first-time parents their only experiences of healthcare related to pregnancy, delivery and postnatal care were from the Swedish healthcare context. However, personal histories, mother and grandmother stories and cultural memories may influence their experiences.

To strengthen the opportunities to participate in the study, the parents could choose if they wanted to be interviewed face-to face or by phone. A majority (*n* = 11) preferred for the interview to be conducted by phone, and the authors didn't ask about the reasons for this. However, it could be seen as a strength, as phone interviews are an efficient and reliable form of collecting data because the risk of power imbalance is less compared to face-to-face interviews (39). The data were analyzed using inductive content analysis. By using this method, the authors had the opportunity to structure the results and present them according to themes. In data interpretation, there is a risk of subjectivity, and text can be interpreted in dissimilar ways. To strengthen the interpretation, the authors had continuous discussions during the analytical procedure. By describing the procedure used and including quotations from the participants, it should be possible for readers to determine the trustworthiness of the study (40). Also, the first and the last author who conducted the interviews have no prior work or research experience within the CHC setting and therefore no preunderstanding when conducting the study.

## Conclusion

The value of continuity from the same midwife who had followed the mother during pregnancy was found important. It was also considered significant that the visits were conducted in the new parents' own home, as that made them feel secure and relaxed. The parents experienced that the knowledge, skills and information of the midwives and the CHC nurses complemented each other and there was a perception of respect toward each other's field of knowledge. By meeting both professionals at the same time made both parents feel safe.

## Data Availability Statement

The datasets presented in this article are not readily available because pursuant to national legislation, ethical review boards in Sweden do not allow release of sensitive raw data to the general public. Requests to access the datasets should be directed to Katarina Sjögren Forss, katarina.sjogren.forss@mau.se.

## Ethics Statement

The study was approved by the Regional Ethical Committee in Lund, Sweden, Approval Number (DNR 2018/841). Written informed consent to participate in this study was provided by the participants' legal guardian/next of kin.

## Author Contributions

KSF and LH carried out the interviews and analyzed the data. KSF wrote the initial version of the manuscript and had feedback from EM and LH. All authors participated in the design of the study. All authors have read and approved the final version of the manuscript.

## Funding

This work was funded by Region Skåne.

## Conflict of Interest

The authors declare that the research was conducted in the absence of any commercial or financial relationships that could be construed as a potential conflict of interest.

## Publisher's Note

All claims expressed in this article are solely those of the authors and do not necessarily represent those of their affiliated organizations, or those of the publisher, the editors and the reviewers. Any product that may be evaluated in this article, or claim that may be made by its manufacturer, is not guaranteed or endorsed by the publisher.
