# Supplementary material for: Interprofessional Teamwork to Promote Health: First-Time Parents' Experiences of a Combined Home Visit by Midwife and Child Health Care Nurse
Source: Front Pediatr. 2022 Mar 3;10:717916. doi: 10.3389/fped.2022.717916 (PMC8927075; doi:10.3389/fped.2022.717916)
Supplement: Supplementary file 1 [file Data_Sheet_1.docx]

**Additional file 1: Interview guide**

1. What expectations did you have on the pediatric healthcare before joining the home visiting program?

2. Can you tell us about your experiences of the home visit?

- was there anything missing?

- How did you perceive meeting both a midwife and a pediatric nurse from pediatric healthcare?

3. Was the home visit as you expected?

4. How would you like the support from the pediatric healthcare to be for you and your family in the future?
