# Supplementary material for: Interprofessional Teamwork to Promote Health: First-Time Parents' Experiences of a Combined Home Visit by Midwife and Child Health Care Nurse
Source: Front Pediatr. 2022 Mar 3;10:717916. doi: 10.3389/fped.2022.717916 (PMC8927075; doi:10.3389/fped.2022.717916)
Supplement: Supplementary file 2 [file Data_Sheet_2.docx]

Additional file 2: Consolidated criteria for reporting qualitative studies (COREQ): 32-item checklist

| No. Item | Guide question/Description | Response |
| --- | --- | --- |
| **Domain 1: Research team and reflexivity** |  |  |
| Personal Characteristics |  |  |
| 1. Interviewer/facilitator | Which author/s conducted the interview or focus group? | KSF and LH conducted the interviews. “Data collection and participants” page 6. |
| 2. Credentials | What were the researcher’s credentials? | KSF: BSc, MPH, PhD  EM: RN, PhD  LH: MPH, PhD |
| 3. Occupation | What was their occupation at the time of the study? | KSF: Senior lecturer  EM: Senior lecturer  LH: Senior lecturer |
| 4. Gender | Was the researcher male or female? | Female. |
| 5. Experience and training | What experience or training did the researcher have? | KSF: Public health and health science  EM: Nursing  LH: Public health and health science |
| Relationship with participants |  |  |
| 6. Relationship established | Was a relationship established prior to study commencement? | No. |
| 7. Participant knowledge of the interviewer | What did the participants know about the researcher? | They knew the reasons for doing the research and the affiliations of the researchers. |
| 8. Interviewer characteristics | What characteristics were reported about the interviewer/facilitator? | Reasons and interests in the research topic. |
| **Domain 2: Study design** |  |  |
| Theoretical framework |  |  |
| 9. Methodological orientation and Theory | What methodological orientation was stated to underpin the study? | A qualitative content analysis.  “Analysis”, page 7. |
| Participant selection |  |  |
| 10. Sampling | How were participants selected? | Recruitment of first-time parents for the interviews were made by registered nurses at the four CHC centers who introduced the research study by providing an information letter and collecting phone numbers from interested participants during their first home visit.  “Research design”, page 5. |
| 11. Method of approach | How were participants approached? | The interested participants were then contacted by phone by the researchers to book a time for an interview.  “Research design”, page 5 |
| 12. Sample size | How many participants were in the study? | In total it was 16.  ”Data collection and participants”, page 6. |
| 13. Non-participation | How many people refused to participate or dropped out? Reasons? | No one refused to participate or dropped out. |
| Setting |  |  |
| 14. Setting of data collection | Where was the data collected? | Data was collected by interviews or by telephone. ”Data collection and participants”, page 6. |
| 15. Presence of non-participants | Was anyone else present besides the participants and researchers? | No. |
| 16. Description of sample | What are the important characteristics of the sample? | In three of the interviews, both parents participated, and in the other interviews, it was either the mother or the father who participated. The age range for the participating families were, for mothers, 15–33 (M=26), and for fathers, 27–37 (M=31). Nine of the interviews were conducted in Swedish, two in English, and two were conducted with the help of an interpreter.  ”Data collection and participants”, page 6. |
| Data collection |  |  |
| 17. Interview guide | Were questions, prompts, guide provided by the authors? Was it pilot tested? | The interview guide was provided by the authors. One pilot interview was held to assure the validity of the questions according to the study’s aim. No changes were warranted, and the pilot interview was included in the analysis. The interview guide is enclosed with the manuscript. ”Data collection and participants”, page 6. |
| 18. Repeat interviews | Were repeat interviews carried out? If yes, how many? | No. |
| 19. Audio/visual recording | Did the researcher use audio or visual recording to collect the data? | The interviews were audiotaped.  ”Data collection and participants”, page 6 |
| 20. Field notes | Were field notes made during and/or after the interview or focus group? | Short field notes were made after the interviews ”Data collection and participants”, page 6. |
| 21. Duration | What was the duration of the interviews or focus group? | The duration of the interviews were 15-30 minutes.  ”Data collection and participants”, page 6 |
| 22. Data saturation | Was data saturation discussed? | Yes.  ”Data collection and participants”, page 6 |
| 23. Transcripts returned | Were transcriptions returned to participants for comments and/or correction? | No. |
| **Domain 3: analysis and findings** |  |  |
| Data analysis |  |  |
| 24. Number of data coders | How many data coders coded the data? | The first and the last author separately read and analysed the text during the process and then discussed it to enhance the best possible account of the meaning found in the texts. “Analysis” page 7. |
| 25. Description of the coding tree | Did authors provide a description of the coding tree? | No. |
| 26. Derivation of themes | Were themes identified in advance or derived from the data? | The themes were derived from the data. “Analysis”, page 7. |
| 27. Software | What software, if applicable, was used to manage the data? | Not applicable. |
| 28.Participant checking | Did participants provide feedback on the findings? | No. |
| Reporting |  |  |
| 29. Quotations presented | Were participant quotations presented to illustrate the themes/findings? Was each quotation identified? | Quotations were presented to illustrate the themes/findings. The quotations are not identified in the paper.  “Results”, page 7-12. |
| 30. Data and findings consistent | Was there consistency between the data presented and the findings? | Yes. “Results”, page 7-12. |
| 31. Clarity of major themes | Were major themes clearly presented in the findings? | Yes.  “Results”, page 7-12. |
| 32. Clarity of minor themes | Is there a description of diverse cases or discussion of minor themes? | Yes “Discussion” page 12-17. |

Developed from : Tong A, Sainsbury P, Craig J. Consolidated criteria for reporting qualitative research (COREQ): a 32-item checklist for interview and focus groups. *Int J Qual Health Care* 2007 19 (6) 349-357.
